# Maternal lifestyle characteristics during pregnancy, and the risk of obesity in the offspring: a study of 5,125 children

**DOI:** 10.1186/s12884-015-0498-z

**Published:** 2015-03-21

**Authors:** Stamatis P Mourtakos, Konstantinos D Tambalis, Demosthenes B Panagiotakos, George Antonogeorgos, Giannis Arnaoutis, Konstantinos Karteroliotis, Labros S Sidossis

**Affiliations:** Department of Nutrition and Dietetics, School of Health Science and Education, Harokopio University, Athens, Greece; Department of Physical Education and Sport Science, University of Athens, Athens, Greece; Department of Internal Medicine, Sealy Center on Aging, Institute for Translational Sciences and Shriners Hospital for Children, University of Texas Medical Branch at Galveston, 301 University Blvd, Galveston, TX USA

## Abstract

**Background:**

To investigate the association between gestational weight gain, maternal age and lifestyle habits (e.g., physical activity, smoking, and alcohol consumption) during pregnancy, with Body Mass Index of the offspring at the age of 8.

**Methods:**

Α random sample of 5,125 children was extracted from a national database and matched with their mothers. With the use of a standardised questionnaire, telephone interviews were carried out for the collection of information like: maternal age at pregnancy, gestational weight gain (GWG), exercise levels, smoking and alcohol consumption. The Body Mass Index (BMI) status of the offspring at the age of 8 was calculated from data retrieved from the national database (e.g., height and weight).

**Results:**

The odds for being overweight/obese at the age of 8 for 1 kg GWG, for smoking, and for mild exercise during pregnancy compared to sedentary was 1.01 (95%CI: 1.00, 1.02), 1.23 (95%CI: 1.03, 1.47) and 0.77 (95%CI: 0.65, 0.91), respectively. Further analysis revealed that offspring of women who exceeded the Institute of Medicine (IOM) maternal weight gain recommendations were at an increased risk of obesity (OR: 1.45; 95%CI, 1.26, 1.67) compared with offspring of women with GWG within the recommended range. Maternal age and alcohol consumption were not associated with the outcome (p > 0.05).

**Conclusion:**

GWG, physical activity and smoking status during pregnancy were significantly associated with obesity for the offspring at the age of 8. Health care professionals should strongly advise women to not smoke and to perform moderate exercise during pregnancy to prevent obesity in the offspring in later life.

## Background

At the dawn of the 21st century the larger part of humanity faces two major epidemics: the sedentary lifestyle and the obesity epidemic. These two usually co-exist, act synergistically to affect the health of individuals, and affect people regardless of sex and age [1]. Among the countries of the European Union, more than half of the adult population is classified as overweight or obese, based on their Body Mass Index (BMI) ≥ 25 [2].

Obesity during pregnancy increases the risk for adverse outcomes both in maternal and offspring health (e.g., pre-eclampsia, gestational diabetes, hypertension, birth by caesarean section, etc.) [3,4]. These are significant concerns for women who are obese at the time of conception, but health risks increase dramatically as mothers gain excessive weight during pregnancy. The extra weight gained during pregnancy may remain after successive pregnancies and it is possibly related to adverse outcomes in the health of the mother [5]. According to the 2009 Institute of Medicine (IOM) recommendations, about 42% of women began pregnancy in 2004–2007 as overweight or obese and 51.2% gained excessive weight (>23 kg) during their pregnancy [6]. These findings along with the increasing incidence of obesity suggest that problems associated with the aforementioned parameters will also emerge in the future.

Researchers that have conducted observational studies report an association of childhood obesity with specific characteristics of pregnancy such as maternal obesity before pregnancy and gestational weight gain (GWG) [7-10]. GWG has also been associated with greater offspring BMI in childhood and early adulthood [11].

Childhood obesity is a worldwide epidemic [12,13]. The number of overweight children is expected to rise by 1.3 million per year, with more than 300,000 of these children becoming obese each year [2]. Greece is among the European countries with the highest levels of childhood obesity and studies report a dramatic increase in its prevalence (52%) [14]. Moreover, in the last decade a significant increase was observed, (~30%), in the rates of overweight 8 to 9 year old children of both genders in Greece [15-17].

Maternal health before and during pregnancy and perinatal factors such as physical activity, smoking and alcohol consumption during pregnancy may play an important role in the health, development, and BMI status of the offspring in the future [18]. Many prospective studies show that childhood obesity is associated with specific characteristics of pregnancy, such as maternal obesity, GWG, birth weight, pregnancy smoking status, alcohol consumption, gestational diabetes [7], and breast feeding [11].

Although obesity in childhood and preadolescence is increasing with alarming rates, long-term epidemiological data that investigate the link between maternal characteristics during pregnancy and obesity status of their offspring in childhood and preadolescence are limited. Current evidence suggests that GWG, smoking and alcohol consumption, as well as exercise during pregnancy independently could be associated with the development of childhood obesity [7,19]. However, no study has ever examined the effect of all the aforementioned variables taken together. Thus, the aim of the present study was to determine how maternal age, GWG, exercise levels during pregnancy, alcohol consumption and smoking are related to obesity of the offspring in preadolescence (e.g., 8 years). The findings of this analysis will provide information on appropriate interventions during pregnancy that could potentially prevent childhood and preadolescence obesity.

## Methods

### Study design

Population-based data, derived from 11 national school-based health surveys, were obtained from a database that included anthropometric data (e.g., weight, height, etc.), as well as contact details of almost all Greek children who attended primary school during 1997–2007, with the exception of 2002 (e.g., total sample 671,715 primary school pupils, aged 7–9 years old), following an official request to the Greek Ministry of Culture and the Ministry of Education. The national database included anthropometric data and information on age, gender, city and area, home address and telephone number, which were collected yearly, at the same time period (spring), from 1997 to 2007, with the exception of 2002, in almost all schools of Primary Education (roughly 85%); schools that did not participate were from borderland areas, with small numbers of children. Thus, from 1997 to 2007, a total of 651,582 8- to 9-year-old children (51% boys and 49% girls, over 95% of the total student population) participated in the study. Measurements were performed by two trained Physical Education (PE) teachers in each school. PE teachers followed a specific protocol taught in corresponding seminars held by the Greek General Secretariat of Sports (GSS). The same protocol was employed in all schools.

### Data extraction

A sample of 5,500 children (0.8% of the entire population) was randomly extracted from the database and their mothers were contacted by telephone. Random extraction was performed through statistical software. The number of 5,500 subjects was adequate to achieve statistical power greater or equal to 99% for evaluating a 0.10 ± 0.05 change in the regression coefficients at 5% significance level of two-sided tested hypotheses. The random sampling was stratified according to the region and place of living (e.g., rural/urban), according to the National Statistical Agency and equally distributed during the study period (i.e., 500 mothers per year). The women that refused to participate in the study were 183 (3.3%). The sample of mother-child dyads covered all geographical regions of Greece (e.g., mainland Greece and the islands). All mothers had Greek nationality.

The information of the proposed protocol was collected through telephone interviews based on the Computer Aided Telephone Interviews (CATI) method. In order to validate the process, 100 face-to-face interviews were conducted to check for discrepancies with the information collected by telephone. No such discrepancies were noted in any of the variables evaluated.

### Measurements

All the necessary information was collected using a standardised questionnaire, named the Childhood Obesity Pregnancy Determinants (ChOPreD) questionnaire, designed and developed with the collaboration of the Harokopeio University Department of Nutrition & Dietetics and Department of Geography and the University of Texas Medical Branch Department of Internal Medicine. The ChOPreD questionnaire was tested and internally revised by study’s investigators during a pilot study, which confirmed its construct validity.

During data collection, the mothers were asked to provide information contained in their pregnancy ultrasound records (e.g., body weight) and recall certain information (e.g., exercise levels, smoking patterns and alcohol consumption). Mothers in Greece have ultrasounds at the start of the pregnancy and several times during its progress and receive records of the results. Only mothers that had full set of records were included in the study, which finalised the sample of 5,125 mother-children dyads. Data recall relating to the perinatal period is very common in pregnancy-related studies.

The BMI data for the children was calculated based on data retrieved from the national database. The BMI status of the offspring at the age of 2 and 8 was determined based on cut-off points suggested by Cole [20]. GWG was calculated based on the difference between the mother’s weight at the last and first visits, based on ultrasound records. Relative GWG was calculated based on the difference between last and first visit compared to first visit.

For the purposes of the current study, physical activity is defined as any form of bodily movement produced by skeletal muscles that increases energy expenditure over the level of physical rest, thereby offering numerous benefits for the human body. This can include a wide range of activities, such as leisure activities, participation in organised sports, exercise, physical work, etc. [21]. The assessment of the exercise was based on frequency (e.g., Never 0 times/wk, Rare 1 time/wk, Often 3–6 times/wk, Daily 7 times/wk), and duration (e.g., exercise more than the recommended 30 minutes) of physical activity. The questionnaire did not evaluate intensity, as only mild intensity exercise is recommended during pregnancy [22].

Cardio-respiratory fitness exercises were recorded as aerobic activities, whereas the ones that involved concentric and eccentric contractions of skeletal muscle exercise were classified as resistance activities [23]. The questionnaire took into account activities undertaken during recreation, exercise or sport, as well as daily activities (e.g., activities one does at work, as part of house and yard work, etc.). Mothers were instructed to refer to all domains of physical activity during their pregnancy. If a mother did not participate in any type of activity she was classified as inactive.

### Study approval

The study was approved by the Bioethics Committee of Harokopio University. Oral approval was obtained from all mothers who agreed to participate in the study and written informed consent was obtained from those participants who took part in the validation process of the study.

### Statistical analysis

Continuous variables were presented as mean values and standard deviations (SD) since they were normally distributed (as examined by the use of histographs and P-P plots) and as median and 1st and 3rd quartile. Categorical variables were presented as absolute and relative frequencies. Offspring BMI obesity status (normal weight vs. overweight/obese) and BMI categories for mothers were calculated according to the proposed cut off points suggested by International Obesity Task Force (IOTF). In order to assess the potential effect of the following maternal characteristics: GWG, smoking during pregnancy, alcohol consumption during pregnancy and level of physical exercise on the offspring’s obesity status, binary logistic regression analysis was implemented and odds ratios (OR) with the corresponding 95% confidence intervals (CI) were calculated. Further adjustments were made for the effect of maternal age at pregnancy, birth weight, maternal weight status pre-pregnancy and history of breastfeeding. Prior to that, every possible effect modification between the proposed risk factors and the confounders was examined, but all interaction terms were not statistical significant (p-values > 0.05). The Hosmer and Lemeshow’s goodness-of-fit test was calculated in order to evaluate the model’s goodness-of-fit and residual analysis was implicated using the dbeta, the leverage, and Cook’s distance D statistics in order to identify outliers and influential observations. All analyses were performed using the SPSS version 18.0 software for Windows (SPSS Inc., Chicago, IL, USA). Statistical significance level from two-sided hypotheses was set at the 5% level (p ≤ 0.05).

## Results

### Baseline characteristics of mothers and offspring

The characteristics of mothers and their offspring are presented in Table 1. The mothers that did not have full set of data were 192 (3.5%). The mothers that did not want to participate in the study were 183 (3.3%). The mean maternal age at pregnancy was 27.8 (4.7) years -median: 28 years, 1^st^ tertile: 25 years, 3^rd^ tertile :30 years- and the age range was 15 to 48 years. The mean GWG was 14.3 (6.1) kg - median: 13 kg, 1^st^ tertile: 10 kg, 3^rd^ tertile :18 kg- and the range was 5 to 45 kg, while the median relative GWG (over maternal weight at first visit) was 21.7% for the entire sample, 27.9% (1st, 3rd tertile 21.2%, 40.0%) for underweight mothers, 22.0% (17.5%, 30.0%) for normal weight mothers, 18.6% (14.0%, 25.7%) for overweight mothers and 12.9% (9.7%, 18.8%) for obese mothers (data not shown). The majority of mothers started their pregnancy with normal BMI (79.9%), while 3.8% were underweight, 14.8% were overweight and only 2.5% were obese. However, 48.1% of mothers were overweight at the end of the pregnancy, 26.5% were obese and only 25.4% retained a normal BMI. The majority of mothers did not exercise during pregnancy (64.5%), while 16.7% exercised moderately, 13.8% sometimes per week, and only 3.2% often and 1.8% daily. Only 11.5% of mothers smoked during pregnancy, while 9.3% consumed alcohol. The children had an average birth weight of 3.33 kg and the range was 1.20-5.80 kg. The average BMI at the age of 8 (child) was 17.6 (3.01) kg/m^2^. With respect to the BMI status of the children, 7.2% were underweight, 57.2% had normal BMI, 24.2% were overweight and 11.4% were obese.

**Table 1:** **Characteristics of the studied sample of mothers and their offspring**

**Offspring characteristics**	
**Males, n(%)**	2686 (52.4%)
**Females, n(%)**	2439 (47.5%)
**Birth weight, Kg**	3.33 (0.50)
**BMI at age 8 (child), Kg/m** ^**2**^	17.6 (3.01)
**BMI status**	
Underweight	370 (7.2%)
Normal	2932 (57.2%)
Overweight	1240 (24.2%)
Obese	583 (11.4%)
**Maternal characteristics**	
**Maternal age at pregnancy, years**	27.8 (4.7)
**Gestational weight gain (GWG), Kg**	14.3 (6.1)
**Maternal BMI status in first visit, n(%)**	
*Underweight*	194 (3.8%)
*Normal*	4044 (79.9%)
*Overweight*	757 (14.8%)
*Obese*	130 (2.5%)
**Maternal BMI status in last visit, n (%)**	
*Normal*	1302 (25.4%)
*Overweight*	2467 (48.1%)
*Obese*	1356 (26.5%)
**Exercise level**	
*Never*	3303 (64.5%)
*Moderate*	858 (16.7%)
*Sometimes per week*	708 (13.8%)
*Often*	162 (3.2%)
*Daily*	94 (1.8%)
**Smoking status during pregnancy, n (%)**	
*Yes*	588 (11.5%)
*No*	4537 (88.5%)
**Alcohol consumption, n(%)**	
*Yes*	476 (9.3%)
*No*	4649 (90.7%)

*Data are presented as absolute and relative frequencies or mean (SD).*

### Determinants of offspring‘s’ obesity status

Logistic regression analysis was conducted to assess the potential effects of maternal age, GWG, exercise levels, alcohol consumption and smoking on obesity status (e.g., overweight/obese vs. normal) of the offspring at the age of 8. The analysis revealed that 1 kg increase in the GWG (within the observed GWG range values) was associated with 1.014-times higher odds of the offspring being overweight/obese (95%CI: 1.00, 1.02) at the age of 8 years; the OR for smoking during pregnancy was 1.23 (95%CI: 1.03, 1.47); and, the OR for moderate exercise during pregnancy compared to sedentary was 0.77 (95%CI: 0.65 0.91). The observed effects were minimal changed when maternal age at pregnancy, birth weight, maternal weight status pre-pregnancy and history of breastfeeding. Were entered in the model as potential confounders [Table 2]. Additional analysis revealed that the offspring of women who exceeded the IOM maternal weight gain recommendations were at an increased risk of obesity (OR: 1.45; 95%CI: 1.26, 1.67) as compared with offspring of women with adequate GWG. Maternal age and alcohol consumption were not associated with the outcome (p > 0.05). The percentage of macrosomic and underweight babies was small and their inclusion in the study did not affect the statistical significance of the results.

**Table 2:** **Results (OR, 95%CI,** ***p*** **) from logistic regression models that used to evaluate the association of maternal characteristics with offspring BMI status (overweight/obesity vs. normal weight) at the age of 8 years**

**Predictors**	**Univariate models** ^**(1)**^ **OR 95% CI**	***p value***	***Full model*** ^***(2)***^ **OR 95% CI**	***p value***	***Full model, plus confounders*** ^***(3)***^ **OR 95% CI**	***p value***
**GWG, per 1 Kg**	1.015 (1.005-1.025)	0.002	1.014 (1.004-1.024)	0.005	1.012 (1.002-1.022)	0.001
**Smoking status during pregnancy (Yes vs. No)**	1.255 (1.053-1.497)	0.011	1.232 (1.03-1.47)	0.023	1.256 (1.044-1.511)	0.016
**Alcohol consumption during pregnancy (Yes vs. No)**	1.028 (0.845-1.251)	0.78	1.11 (0.90-1.36)	0.33	1.141 (0.924-1.408)	0.22
**Exercise level during pregnancy**		0.041		0.039		0.019
**Moderate vs. sedentary**	0.786 (0.670-0.923)	0.003	0.771 (0.654-0.910)	0.002	0.747 (0.631-0.884)	0.001
**Sometimes per week vs. sedentary**	0.958 (0.809-1.135)	0.620	0.961 (0.809-1.140)	0.646	0.953 (0.800-1.135)	0.59
**Often vs. sedentary**	0.787 (0.561-1.104)	0.166	0.84 (0.593-1.192)	0.329	0.87 (0.606-1.243)	0.44
**Daily vs. sedentary**	0.884 (0.574-1.362)	0.576	0.891 (0.554-1.433)	0.636	0.955 (0.589-1.549)	0.85

^(1)^The effect of each predictor on the offspring BMI status was separately evaluated; ^(2)^Includes all predictors (i.e. maternal age at pregnancy, gestational weight gain, exercise levels, alcohol consumption and smoking on obesity status); ^(3)^Includes all predictors entered in the full model plus, birth weight, maternal weight status pre-pregnancy and history of breastfeeding, as potential confounders.

## Discussion

The aim of the present work was to investigate the association between GWG, maternal age and various lifestyle habits, like physical activity, smoking, and alcohol consumption during pregnancy, with body weight of the offspring at the age of 8. It was revealed that GWG, physical activity and smoking status during pregnancy were significantly associated with obesity for the offspring at the age of 8 years. Moderate exercise during pregnancy was found to lower the risk of the offspring to develop overweight/obesity in childhood and preadolescence, even after adjusting for various maternal and offspring characteristics.

The pregnancy period is a phase in a woman’s life in which she develops a greater awareness about her health. During pregnancy, women are given a significant opportunity to amend some unhealthy habits, like smoking and alcohol consumption, to adopt a more active lifestyle, and to participate in physical activities and/or exercise. The development and introduction of specific recommendations for physical activity for pregnant women is relatively recent. The investigation of physical activity among pregnant women began in the last quarter of the 20th century and continues to this day. More specifically, early investigations in the 1970s and 1980s included a very cautious approach and focused mainly on possible adverse effects for the health of pregnant women, primarily because of the limited knowledge about its response of pregnant women to exercise and the even more limited knowledge about the effects on pregnancy. Only recently, researchers have begun to focus on the potential benefits to the health of mothers and their offspring that are related to participation in exercise during pregnancy. The Guide for Physical Activity in the US for 2008 was a crucial point, as it contained inter alia a well-written and substantiated chapter on the role of physical activity during pregnancy and after it [24,25]. Based on the recommendations proposed in the Guide and the recommendations of other countries, it is suggested that pregnant healthy women can exercise at the same level as non-pregnant women, especially early in the pregnancy. According to evidence gathered from the Behavioural Risk Factor Survey that was conducted in 2000, Evenson and Wen report that more than two thirds of pregnant women said that they participated in some type of leisure physical activity [26]. Since the prevalence of pregnant women’s participation in physical activities is increasing, it is important to understand the potential risks and the possible benefits of physical activity during pregnancy for women and their offspring.

Despite the fact that the benefits of exercise for the wider population have been internationally accepted, the claims for its beneficial effects during pregnancy have not yet been substantiated [27-31] and exercise is not yet sufficiently well accepted as being beneficial for pregnant women. Health scientists are still sceptical and often reluctant to encourage exercise during pregnancy, despite the well-recognised benefits. One of the main concerns associated with exercise during pregnancy is the effect of the activity to the foetus, as any benefits to the mother can be offset by adverse effects to the foetus. Although the concerns are theoretically associated with the selective redistribution of blood flow during exercise and the transport of CO_2_ and O_2_, and nutrients by the placenta, it has been shown that moderate exercise seems to cause minimal to moderate increase in foetal heart rate by approximately 10–30 beats/minute above baseline [28]. On the contrary, physical activity during pregnancy has been shown to improve the health status of both the mother and the foetus. Moreover, maternal exercise may reduce the risk for certain risk factors of pregnancy-related complications, such as gestational diabetes according to many studies [31-34].

In accordance with the guidelines for prenatal physical activity in the United States, the American College of Obstetrics and Gynaecology (ACOG) currently recommends that pregnant women are allowed to undertake 30 or more minutes of moderate exercise on most, if not all days of the week, if there are no health problems and obstetric complications [32]. Recommendations for physical activity from the American Ministry of Health published in 2008, state that pregnant women must participate in at least 150 minutes of moderate-intensity aerobic exercise a week, even if they did not participate in such activities before pregnancy [35]. The American College of Sports Medicine (ACSM) recommends at least 3 sessions of exercise lasting at least 15 minutes and whose duration will increase gradually to 30 minutes a day, preferably all days of the week [36]. The recommendations are similar in Canada [37], Denmark [38], Great Britain [39], Norway [40] and Australia [41].

Another significant risk factor during pregnancy is related to smoking. It is a common and preventable specific adverse environmental exposure for the foetus [42]. Maternal smoking during pregnancy is associated with foetal growth retardation and increased risk of preterm delivery and low birth weight [43,44]. Maternal smoking during pregnancy also seems to increase the risk of obesity in the offspring [45,46].

Regarding smoking during pregnancy, the results from the present study are in line with other studies that show that exposure to smoking during foetal life leads to overweight and obesity in childhood. A systematic review showed that prenatal exposure to maternal cigarette smoking led to a 50% increased risk of overweight at the age of 3–33 years old [47]. Also, a recent meta-analysis showed that maternal smoking during pregnancy was associated with obesity in children with an average age of 9 years [48]. It has also been suggested that there is a dose-response relationship between the number of cigarettes smoked and the risk of childhood obesity [49]. Several studies have also shown an association between maternal smoking during pregnancy with the highest BMI in the offspring or the increased risk of obesity in later life [45,49-51].

Moreover, it has been argued that exposure of the developing foetus to nicotine may adversely affect the development of the function of the hypothalamus and through this mechanism to have an effect on appetite control during later life and consequently to increase the risk of future obesity [52]. Furthermore, studies showed that children of mothers who smoked had a higher BMI at 1 year of age [53], and in separate studies, increased BMI was also evident at the age of 6.5 years [54], 8 years [55], even in 33 years [53].

Finally, another survey [56] recently showed that teenagers in late adolescence that had been exposed to smoking as foetuses showed higher values of subcutaneous fat (26%) and endo-abdominal fat (33%). Overall, while the weight gain in children from the mother’s smoking is small, the results are long-termed.

Several studies in recent years have evaluated the reliability of recalled information relating to the perinatal period. Specifically, studies have been conducted involving recall intervals from 7 to 22 years and on the whole concluded that this information is reliable [57-59].

An earlier survey by Villar et al. (1988) showed high correlation between measured and recalled variables, such as anthropometric measurements of the mother and the offspring, but low correlation to factors such as physical activity during pregnancy and blood pressure [60]. Finally, a review of studies that employed physical activity questionnaires during pregnancy compared to ones that employed objective measurements (e.g. accelerometers) showed that the association between them was low to moderate [22]. Hence the results, as in the present investigation, should be interpreted with caution.

### Limitations

The information that was collected during the telephone interviews was self-reported, and although mothers could provide information based on health records for themselves, this forms a limitation of the study. Moreover, a potential limitation of the study was that in the current cohort 17.3% of women were overweight/ obese before their pregnancy, a relatively low prevalence in comparison to published reports for the corresponding population [61,62]. This could be attributed to deliberate under-reporting, over-reporting or recall bias for the self-reported pre-pregnancy anthropometric data (body weight and height) [63]. Similar observations have been previously reported in Greece by Manios et al., 2009 [64] and is a common limitation in similar studies [65].

Finally, the sample of mothers included in the study did not show statistically significant levels of other risk factors related to intrauterine or foetal growth (i.e., gestational diabetes, increased blood pressure, etc.). Thus, the researchers concentrated their analysis only on the risk associated with GWG, maternal age at pregnancy, alcohol consumption, smoking and exercise.

The authors report no conflict of interest in the reporting of the data.

## Conclusion

The research analysis that was conducted confirmed that when a mother gains more weight, adopts sedentary behaviour and smokes during pregnancy, the risk that her offspring will be overweight or obese (e.g., higher BMI) at the ages of 8 increases significantly.

Health care professionals should advise women to limit their GWG to the range specified for their pre-pregnancy BMI according to IOM guidelines, not to smoke and consume alcohol, and do moderate exercise during pregnancy.
